# Attenuating Effects of Dieckol on High-Fat Diet-Induced Nonalcoholic Fatty Liver Disease by Decreasing the NLRP3 Inflammasome and Pyroptosis

**DOI:** 10.3390/md19060318

**Published:** 2021-05-30

**Authors:** Seyeon Oh, Myeongjoo Son, Kyung-A Byun, Ji Tae Jang, Chang Hu Choi, Kuk Hui Son, Kyunghee Byun

**Affiliations:** 1 Functional Cellular Networks Laboratory, Department of Medicine, College of Medicine, Graduate School and Lee Gil Ya Cancer and Diabetes Institute, Gachon University, Incheon 21999, Korea; seyeon8965@gmail.com (S.O.); mjson@gachon.ac.kr (M.S.); kabyun95@gmail.com (K.-A.B.); 2 Department of Anatomy & Cell Biology, Gachon University College of Medicine, Incheon 21936, Korea; 3 Aqua Green Technology Co., Ltd., Smart Bldg., Jeju Science Park, Cheomdan-ro, Jeju 63309, Korea; whiteyasi@gmail.com; 4 Department of Thoracic and Cardiovascular Surgery, Gachon University Gil Medical Center, Gachon University, Incheon 21565, Korea; cch624@gilhospital.com

**Keywords:** nonalcoholic fatty liver disease, inflammasome, pyroptosis, *Ecklonia cava* extract, dieckol

## Abstract

Nonalcoholic fatty liver disease (NAFLD), which promotes serious health problems, is related to the increase in the nucleotide-binding oligomerization domain-like receptor family, pyrin domain containing 3 (NLRP3) inflammasome and pyroptosis by a high-fat diet (HFD). Whether dieckol (DK), a component of *Ecklonia cava* extracts (ECE), attenuated NAFLD in an HFD-induced NAFLD animal model was evaluated. The expression of high mobility group box 1/Toll-like receptor 4/nuclear factor-κB, which initiated the NLRP3 inflammasome, was increased in the liver of HFD-fed animals and significantly decreased with ECE or DK administration. The expression of NLRP3/ASC/caspase-1, which are components of the NLRP3 inflammasome, and the number of pyroptotic cells were increased by HFD and decreased with ECE or DK administration. The accumulation of triglycerides and free fatty acids in the liver was increased by HFD and decreased with ECE or DK administration. The histological NAFLD score was increased by HFD and decreased with ECE or DK administration. The expression of lipogenic genes (FASN, SREBP-2, PPARγ, and FABP4) increased and that of lipolytic genes (PPARα, CPT1A, ATGL, and HSL) was decreased by HFD and attenuated with ECE or DK administration. In conclusion, ECE or DK attenuated NAFLD by decreasing the NLRP3 inflammasome and pyroptosis.

## 1. Introduction

Nonalcoholic fatty liver disease (NAFLD) is categorized by histology, pathogenesis, and natural history from isolated steatosis or nonalcoholic fatty liver to nonalcoholic steatohepatitis (NASH) [1]. Isolated steatosis is characterized by excess fat deposition without injury or inflammation; however, NASH is accompanied by hepatocyte ballooning, liver injury, inflammation, and varying degrees of fibrosis, eventually causing cirrhosis and acting as risk factors of end-stage liver disease and hepatocellular carcinoma [1].

NAFLD prevalence is predicted to be between 20% and 30% in the general population, but it is increasing by up to 75% in those with morbid obesity [2,3,4].

The definite etiology of NAFLD has not been fully revealed. It has been suggested that dietary fat has an essential role in the development or progression of hepatic steatosis and NASH in humans [5,6]. Animal models fed with a high-fat diet (HFD) have shown histological changes and metabolic abnormalities of NAFLD [7,8,9].

Many studies have reported that pyroptosis or the nucleotide-binding oligomerization domain-like receptor family, pyrin domain containing 3 (NLRP3) inflammasome, is involved in the development or progression of NAFLD [10].

Pyroptosis is cell death by pore formation induced by caspase-1/4/5/11 in the cell membrane after the release of proinflammatory mediators such as interleukin (IL)-18/1β [11]. The characteristic features of pyroptosis are cell swelling, increased permeability of the cell membrane, cell lysis, and release of cytoplasmic content or proinflammatory mediators [11]. In NAFLD, lipotoxic hepatocytes secrete high mobility group box 1 (HMGB1), a damage-associated molecular pattern (DAMP), as a response to inflammation [12,13]. Extracellular HMGB1 binds to its receptors, such as Toll-like receptor 4 (TLR4), which aggravates liver inflammation and cell death [12,13]. TLR4 signal pathways increase nuclear factor-κB (NF-κB) expression, which eventually increases the synthesis of NLRP3 [14,15]. When NLRP3 binds to an apoptosis-associated speck-like protein containing a caspase recruitment domain (ASC) and pro-caspase-1, the NLRP3 inflammasome is assembled [16,17]. The NLRP3 inflammasome activates caspase-1, consequently leading to the maturation and excretion of IL-1β and IL-18 [16,17]. Activated caspase also increases pyroptosis by dissociating gasdermin D, which forms pores in the plasma membrane [18].

*Ecklonia cava,* which is a brown seaweed found on the coastline of Korea, showed various beneficial effects such as anti-inflammatory [19,20], anti-oxidant [21], and anti-adipogenic effects [22]. In addition, phlorotannins from *Ecklonia cava* have been reported to have various effects, such as decreasing inflammation, which is induced by HFD [23,24]. Dieckol (DK)-enriched extraction from Laminaria japonica has been reported to decrease hepatic steatosis by stimulating hepatic fatty acid β-oxidation [25].

However, it has not been revealed whether DK, a phlorotannin from *Ecklonia cava*, attenuated NAFLD. In this study, the effects of *Ecklonia cava* extract (ECE) and DK on NAFLD were evaluated by decreasing the formation of the NLRP3 inflammasome and pyroptosis in an HFD-induced mouse NAFLD model.

## 2. Results

### 2.1. ECE and DK Decreased HMGB1, TLR4, and NF-κB Expression in the Liver of HFD Mice

**Figure 1:**
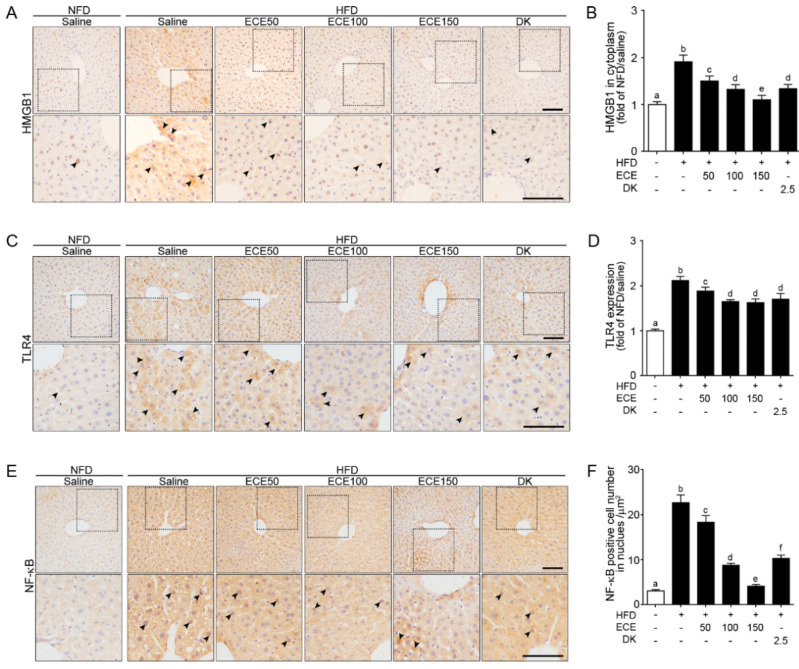
Effects of ECE and DK on HMGB1, TLR4, and NF-κB expression in the liver of the HFD-induced NAFLD mouse model. (**A**–**F**) In liver tissue, the HMGB1 (**A**), TLR4 (**C**), and NF-κB (**E**) protein levels were increased by HFD/saline. The addition of ECE and DK decreased the protein levels of HMGB1, TLR4, and NF-κB. Quantified graphs showed HMGB1 expression in cytoplasm (**B**), TLR4 intensity in whole cell (**D**), and NF-κB-positive cell numbers in the nucleus (**F**). Data are mean ± SD. *p* < 0.05; a–f; Same letters indicate nonsignificant differences between groups as determined by multiple comparison (Mann–Whitney U test). DK, dieckol; ECE, *Ecklonia cava* extract; HFD, high-fat diet; HMGB1, high mobility group box 1; NFD, normal fat diet; NF-κB, nuclear factor kappa-light-chain-enhancer of activated B cells; TLR4, Toll-like receptor 4.

The HMGB1 expression in the cytoplasm was significantly increased in the liver of the HFD group compared to in the NFD group and significantly decreased with either ECE or DK administration. The decreasing effect was most prominent in 150 mg/kg ECE (Figure 1A,B). The TLR4 expression was significantly increased in the liver of the HFD-fed mouse compared to in the NFD-fed mouse and significantly decreased with either HFD fed mouse with ECE or DK. The decreasing effect was most prominent in 100 mg/kg, 150 mg/kg ECE, and 2.5 mg/kg DK (Figure 1C,D). The number of NF-κB-positive cells in the nuclei was significantly increased in the liver of the HFD group compared to in the NFD group and significantly decreased with either ECE or DK administration. The decreasing effect was most prominent in 150 mg/kg ECE (Figure 1E,F).

### 2.2. ECE and DK Attenuated the NLRP3 Inflammasome and Pyroptosis in the Liver of HFD Mice

**Figure 2:**
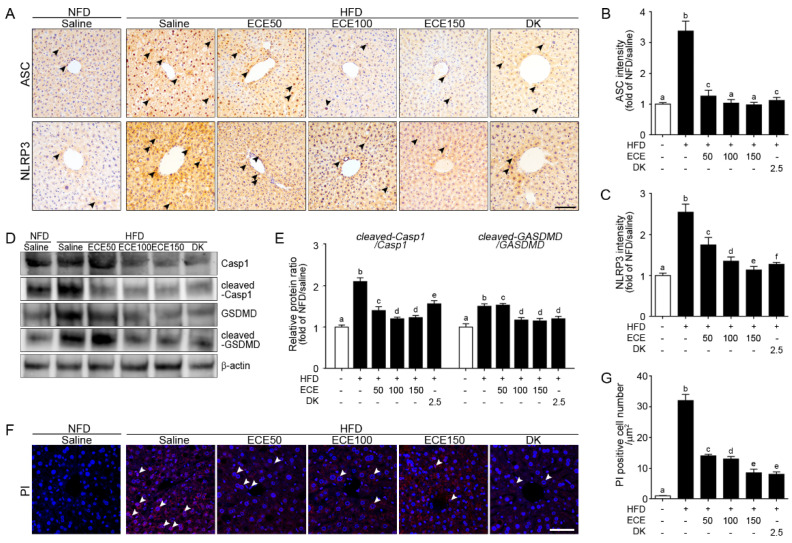
Effects of ECE and DK on the NLRP3 inflammasome-induced pyroptosis in the liver of the HFD-induced NAFLD mouse model. In liver tissue, (**A**–**C**) the ASC (upper row of **A** and **B**) and NLRP3 (lower row of **A** and **C**) expression levels were increased by HFD/saline and decreased by ECE or DK treatment. Scale bar, 200 µm. (**D** and **E**) Immunoblotting results show expression of Casp1, cleaved-Casp1, GSDMD, and cleaved-GSDMD. (**F** and **G**) The PI-positive cell numbers in the liver were increased by HFD/saline and decreased after treatment with ECE or DK. Data are mean ± SD. *p* < 0.05; **a**–**f**; Same letters indicate nonsignificant differences between groups as determined by multiple comparison (Mann–Whitney U test). ASC, apoptosis-associated speck-like protein containing a CARD; Casp1, caspase-1; DK, dieckol; ECE, *Ecklonia cava* extract; GSDMD, gasdermin D; HFD, high-fat diet; NFD, normal fat diet; NLRP3, NOD-like receptor family, pyrin domain containing 3; PI, propidium iodide.

ASC expression was significantly increased in the liver of the HFD group compared to in the NFD group and significantly decreased with either ECE or DK administration. The decreasing effect was most prominent in 100 mg/kg and 150 mg/kg ECE (Figure 2A,B).

NLRP3 expression was significantly increased in the liver of the HFD group compared to in the NFD group and significantly decreased with either ECE or DK administration. The decreasing effect was most prominent in 150 mg/kg ECE (Figure 2A,C).

Expression ratio of caspase-1 and cleaved-caspase-1 was significantly increased in the liver of the HFD group compared to in the NFD group and significantly decreased with either ECE or DK administration. The decreasing effect was most prominent in 100 mg/kg and 150 mg/kg ECE (Figure 2D,E). Expression ratio of gasdermin D (GSDMD) and cleaved-GSDMD was significantly increased in the liver of the HFD-fed mouse compared to in the NFD-fed mouse and significantly decreased with either HFD mouse fed with ECE or DK. The decreasing effect was most prominent in 100 mg/kg, 150 mg/kg ECE, and 2.5 mg/kg DK (Figure 2D,E). Cells that underwent pyroptosis were stained by PI [26]. The number of PI-positive cells in the HFD group was significantly higher than that in the NFD group and significantly decreased with either ECE or DK administration. The decreasing effect was most prominent in 150 mg/kg ECE and 2.5 mg/kg DK (Figure 2F,G).

### 2.3. ECE and DK Decreased NAFLD Activity in HFD Mice

**Figure 3:**
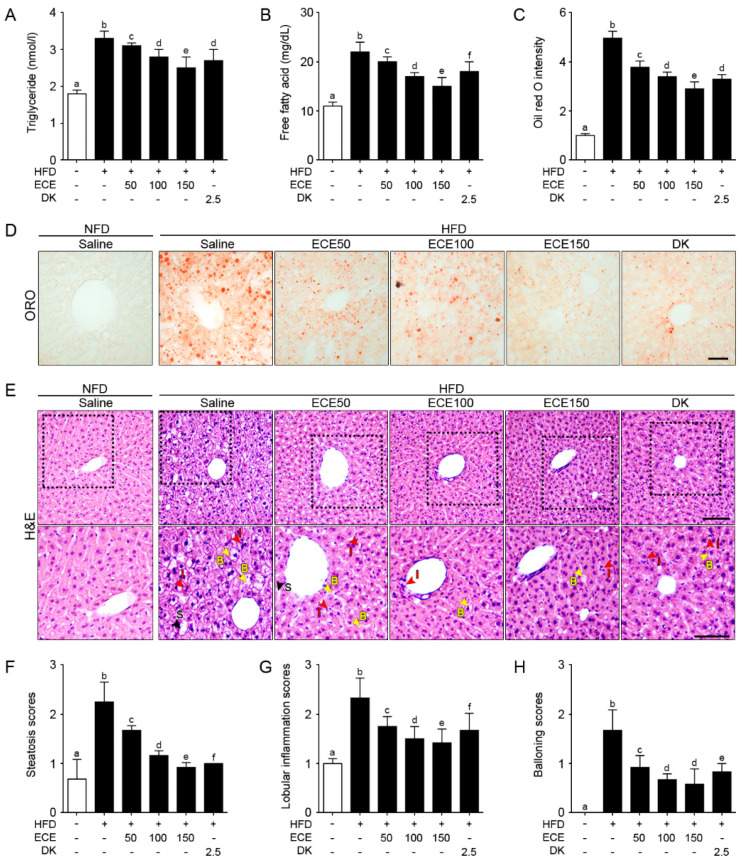
Effects of ECE and DK on controlling lipid deposition and NAFLFD activity. (**A** and **B**) Triglycerides (**A**) and free fatty acids (**B**) in the liver were increased by HFD/saline and decreased by ECE or DK treatment. (**C** and **D**) The hepatic lipid deposition by ORO staining was increased by HFD/saline and decreased after treatment with ECE or DK. (**E**–**H**) The NAFLD activity was scored by H&E staining (upper row). Enlarged images (lower row) show hepatic steatosis conditions including steatosis (S; black arrows), inflammation (I; red arrows), and ballooning (**B**; yellow arrows) of liver (**E**). Quantified graphs showed that the hepatic steatosis score (**F**), lobular inflammation score (**G**), and ballooning score (**H**) were increased by HFD/saline and decreased after treatment with ECE or DK. Data are mean ± SD. *p* < 0.05, **a**–**f**; Same letters indicate nonsignificant differences between groups as determined by multiple comparison (Mann–Whitney U test). DK, dieckol; ECE, *Ecklonia cava* extract; HFD, high-fat diet; H&E, hematoxylin and eosin staining; ORO, oil red O staining; NFD, normal fat diet.

The triglyceride level was significantly increased in the liver of the HFD group compared to in the NFD group and significantly decreased with either ECE or DK administration. The decreasing effect was most prominent in 150 mg/kg ECE (Figure 3A). The free fatty acid level was significantly increased in the livers of the HFD group compared to in the NFD group and significantly decreased with either ECE or DK administration. The decreasing effect was most prominent in 150 mg/kg ECE (Figure 3B). The steatosis area evaluated by ORO intensity was significantly increased in the livers of the HFD group compared to in the NFD group and significantly decreased with either ECE or DK administration. The decreasing effect was most prominent in 150 mg/kg ECE (Figure 3C,D).

Macrovesicular steatosis, lobular inflammation, and hepatocellular ballooning evaluated by H&E staining in the liver [27,28,29] of the HFD group were significantly increased compared to in the NFD group and significantly decreased with either ECE or DK administration. The decreasing effect was most prominent in 150 mg/kg ECE (Figure 3E–H).

### 2.4. ECE and DK Reduced Lipogenesis and Increased Lipolysis in the Liver of HFD Mice

**Figure 4:**
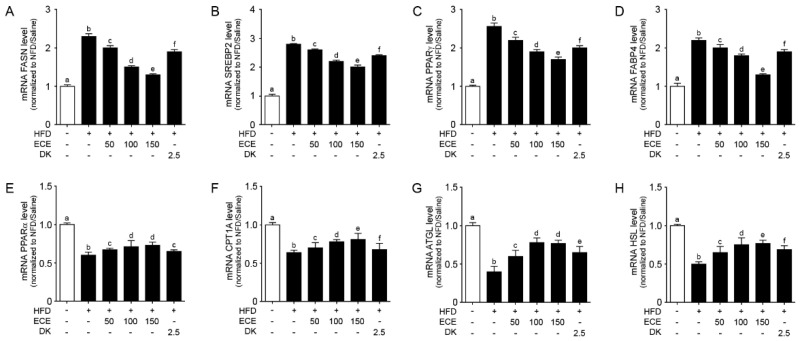
Effects of regulating lipid synthesis and oxidative genes in the liver of the HFD-induced NAFLD mouse model due to the regulation of NAFLD activity. In liver tissue, (**A**–**D**) the lipogenesis related to gene mRNA levels, including FASN (**A**), SREBP2 (**B**), PPARγ (**C**), and FABP4 (**D**), were increased by HFD/saline. The addition of ECE and DK decreased those mRNA levels. (**E**–**H**) The lipolysis related to gene mRNA levels, including PPARα (**E**) CPT1A (**F**), ATGL (**G**), and HSL (**H**), were decreased by HFD/saline and decreased by ECE or DK treatment. Data are mean ± SD. *p* < 0.05, **a**–**f**; Same letters indicate nonsignificant differences between groups as determined by multiple comparison (Mann–Whitney U test). ATGL, adipose triglyceride lipase; CPT1A, carnitine palmitoyltransferase 1A; DK, dieckol; ECE, *Ecklonia cava* extract; FABP4, fatty acid-binding protein 4; FASN, fatty acid synthase; HFD, high-fat diet; HSL, hormone sensitive lipase; NFD, normal fat diet; PPARα, peroxisome proliferator-activated receptor alpha; PPARγ, peroxisome proliferator-activated receptor gamma; SREBP2, sterol regulatory element-binding protein 2.

The expression of lipogenic genes (FASN, SREBP2, PPARγ, and FABP4) [30,31] was significantly increased in the liver of the HFD group compared to in the NFD group and significantly decreased with either ECE or DK administration. The decreasing effect was most prominent in 150 mg/kg ECE (Figure 4A–D). 

The expression of lipolytic genes (PPARα, CPT1A, ATGL, and HSL) [30,32] was significantly decreased in the liver of the HFD group compared to in the NFD group and significantly increased with either ECE or DK administration. The increasing effect was most prominent in 150 mg/kg ECE (Figure 4E–H).

## 3. Discussion

Lipid levels in the liver are controlled by balancing various processes, such as absorption, synthesis, oxidation, and lipid exports. When the input of fatty acids to hepatocytes by either ingestion or synthesis is higher than the output of fatty acids by oxidation or export, lipid accumulation is aggravated in the liver [33,34]. HFD is a risk factor for the development of NAFLD in humans, and numerous HFD animal models showed that HFD promotes NAFLD [35,36].

Excessive free fatty acids from HFD or released from adipose tissue are absorbed by the liver [37]. Fatty acids are also generated in the liver by de novo lipogenesis [38]. Increased free fatty acids lead to lipotoxic processes, including death receptor signaling, initiation of endoplasmic reticulum stress, mitochondrial apoptosis, activation of Toll-like receptors, assembly of inflammasomes, and autophagy blockage [39,40].

Among these, the NLRP3 inflammasome has received extra attention as an essential mechanism of NAFLD or NASH [10]. The NLRP3 inflammasome is activated by the release of ATP from necrotic cells, HMGB1, histones, amyloid, and uric acid crystals [41,42,43,44].

Several studies have shown the HMGB1-dependent pathway of lipotoxic hepatocyte injuries in the early stages of NASH [12,45]. TLR4 by binding to HMGB1 induces NLRP3 inflammasome activation in NASH [12,45]. The NLRP3 inflammasome consists of three parts: a receptor protein (NLRP3), an adaptor protein (ASC), and an effector protein (caspase-1) [46,47]. The receptor protein is a sensor for the pathogen-associated molecular pattern (PAMP) or DAMP and is switched on after sensing. The ASC adaptor protein has two death domains, the N-terminal pyrin domain and the C-terminal caspase recruitment domain (CARD), and acts as a mediator between the sensor and the effector protein [47,48]. NLRP3 activation goes through two steps of priming and activation [49]. The priming signal starts from the activation of pattern recognition receptors, such as TLR, by PAMP or DAMP, which activates the NF-κB pathway. These signals increase the transcription and expression of NLRP3 and the translocation of pro-IL-1β and pro-IL-18 from the nucleus to the cytoplasm [50]. The activating signal, which initiates the stimulation of the NLRP3 inflammasome, is initiated by various activators, such as PAMPs, DAMPs, exogenous adenosine, mitochondrial DNA, or substances [51,52,53]. Consequently, ASC via CARD recruits pro-caspase-1 and promotes inflammasome assembly. The activated NLRP3 inflammasome changes pro-caspase-1 to mature caspase-1 by autocleavage. The mature caspase-1 initiates the cleavage of the precursor cytokines pro-IL-1 β and pro-IL-18 into their mature forms of IL-1β and IL-18 [54,55].

Furthermore, caspase-1 leads to cleavage of GSDMD into two fragments: C-terminal domain and N-terminal domain. The N-terminal domains move to the plasma membrane and make cell membrane pores [18,55].

This study showed that HFD increased HMGB1 expression in the liver. Moreover, TLR4 and NF-κB expression was increased by HFD in the liver. These increases were decreased with ECE or DK administration. The NLRP3 inflammasome components, such as ASC and NLRP3, were increased in the liver of HFD mice and decreased with ECE or DK administration. The ratio of cleaved-caspase-1 and caspase-1 was increased by HFD in the liver, and it was decreased by ECE or DK administration. The ratio of cleaved-GSDMD and GSDMD was increased by HFD in the liver, and it was decreased by ECE or DK administration.

Pyroptosis is characterized by cytoskeletal rearrangement, pore formation in the plasma membrane, DNA fragmentation, and release of proinflammatory cytokines [56]. Small molecular weight dyes, such as PI, enter the cytosol through pores in the plasma membrane of pyroptotic cells, whereas the apoptotic cell membrane is intact and cannot be stained by PI [26,57]. Several studies have shown that HFD induces increasing pyroptosis in the liver [58].

In this study, PI-stained cells were increased in the liver of HFD mice and significantly decreased with ECE or DK administration. The triglyceride and free fatty accumulation in the liver of HFD mice were higher than in NFD.

Previous studies showed that HFD induced increased hepatic TG and steatosis [27,59]. Here, HFD increased hepatic TG and FFA, and they were decreased by administration of ECE or DK. 

The histological changes in macrovesicular steatosis, lobular inflammation, and hepatocellular ballooning in the liver of HFD mice were more severe in HFD mice than in NFD mice. These changes were attenuated with ECE or DK administration. Increased FASN, SREBP2, PPARγ, and FABP4 [30,31] aggravated the accumulation of cholesterol or triglycerides in the liver and was involved in lipid toxicity [60,61,62,63]. Meanwhile, PPARα, ATGL, and HSL [30,32] increased CPT1A expression and promoted β-oxidation and decreased expression of FASN, inhibiting lipid accumulation [64]. In this study, FASN, SREBP2, PPARγ, and FABP4 expression was increased by HFD in the liver and decreased with either ECE or DK administration. However, PPARα, CPT1A, ATGL, and HSL expression was decreased in the liver of the HFD group compared to in the NFD group and increased with either ECE or DK administration. It seems that ECE or DK inhibited lipid accumulation in the liver by increasing lipolysis and decreasing lipogenesis.

It is known that saturated FFA induces hepatocyte apoptosis by various mechanisms such as endoplasmic reticulum stress, death receptor and c-Jun N-terminal kinase signaling, reactive oxygen species, non-coding RNAs, and dysregulation of autophagy [65]. These signal pathways consequently lead to mitochondrial dysfunction, which results in cell death [65].

Our study showed that increasing pyroptosis by HFD also led to NAFLD, and ECE or DK could decrease development of NAFLD by decreasing pyroptosis.

## 4. Materials and Methods

### 4.1. ECE and DK Preparation

ECE and DK preparation methods were followed from a previous study [66]. Briefly, E. cava raw material was washed thoroughly with water and dried for 2 days. The E. cava material was finely ground, a 10-fold volume of 50% alcohol was added, and then it was boiled to 85 °C for 12h and finally spray-dried. 

Dieckol (DK), which is a representative phlorotannin of ECE, was then isolated from ECE. Centrifugal partition chromatography was conducted using a two-phase system that mixed n-hexane, methanol, pure water, and ethyl acetate (v/v/v/v 7:7:2:3). The organic stationary phase and the mobile phase of the column were filled, following the descending order of flow rate (2 mL/min), and were used for isolation. The DK structure and spectrogram can be found in Oh et al. [66]. 

### 4.2. HFD-Induced NAFLD Mice Model

C57BL/6N male mice (7 weeks old; Orient Bio, Sungnam, Korea) were maintained at a controlled condition (temperature of 23 °C with 50% humidity, under 12 h light/12 h dark cycle).

The HFD induction model is widely known as the NAFLD animal model [67]. After 7 days of the adaptation period, mice were randomly divided into six groups: mice in Group 1 were fed a normal fat diet (NFD) ad libitum for 4 weeks and then 0.9% normal saline by oral administration for 4 weeks (Group 1; NFD/saline). Mice in Groups 2 to 6 were fed 45% HFD (Research Diet, Inc., New Brunswick, NJ, USA) for 4 weeks. After 4 weeks on HFD, mice were orally co-administered with 0.9% normal saline (Group 2; HFD/saline), ECE (Group 3: 50 mg/kg/day; HFD/ECE50, Group 4: 100 mg/kg/day; HFD/ECE100, and Group 5: 150 mg/kg/day; HFD/ECE150), which is an isolation method described in a previous study [66], or DK (Group 6: 2.5 mg/kg/day; HFD/DK) for 4 weeks.

Eight weeks after treatment commencement, liver samples were collected in accordance with ethical principles issued, and the study was approved by the Institutional Animal Care and Use Committee of Gachon University (approval no. LCDI-2019-0130).

### 4.3. Isolation of RNA and Quantitative Real-Time-Polymerase Chain Reaction (qRT-PCR)

Frozen liver tissues (50 mg) were ground using mortar containing liquid nitrogen and then homogenized in 500 µL RNiso (Takara, Kusatsu, Japan). Homogenates were mixed with 100 µL chloroform and centrifuged at 12,000 × g for 15 min at 4 °C. The aqueous layers were collected in cleaned tubes, mixed with 250 µL isopropanol, and centrifuged using the same conditions. Isolated RNA samples were washed with 500 µL of 75% ethanol and dissolved in 30 µL diethyl pyrocarbonate-treated water. For qRT-PCR, RNA was converted to cDNA using a Prime Script First Strand cDNA Synthesis Kit (Takara, Kusatsu, Japan). After synthesis, qRT-PCR was performed using the CFX 384 Touch™ Real-Time PCR detection system. The reaction efficiencies and cycle threshold numbers were determined using the CFX Manager™ software 3.1. For internal control, β-actin was used, and the primer sequences for target genes are detailed in Table S1.

### 4.4. 3,3′-Diaminobenzidine (DAB) Staining Immunohistochemistry

Liver paraffin tissue slides (7 µm) were deparaffinized and rehydrated. The deparaffinized slides were treated with 0.3% H_2_O_2_ (Sigma-Aldrich, St. Louis, MO, USA) for H_2_O_2_ block and then washed in phosphate-buffered saline (PBS). The slides were incubated in animal serum to block a nonspecific background, applied with primary antibodies (as listed in Table S2), and then rinsed three times with PBS. The probed slides were treated with biotinylated secondary antibodies from the ABC kit (Vector Laboratories, Burlingame, CA, USA), incubated for 1 h in blocking solution, and washed three times using PBS. The slides were developed with DAB substrate for 5 to 15 min, incubated with hematoxylin to cover the section, and then mounted with a coverslip using DPX mounting solution (Sigma-Aldrich, St. Louis, MO, USA). The images were visualized by light microscopy (Olympus Optical Co., Tokyo, Japan), and quantification of the intensity of the brown color was performed using ImageJ software 1.53j (National Institutes of Health, Bethesda, MD, USA).

### 4.5. Protein Extraction and Immunoblotting

Fifty milligrams of frozen liver tissues was ground using mortar with liquid nitrogen and then incubated for 15 min on an ice container with added RIPA lysis buffer (EzRIPA; ATTO, Tokyo, Japan), mixed proteinase, and phosphatase inhibitors. After they were centrifuged at 14,000× *g* for 15 min at 4 °C, the supernatant was collected in a new tube, and the protein concentration was analyzed using a bicinchoninic acid assay kit (BCA kit; Thermo Fisher Scientific, Inc., Waltham, MA, USA). Equal amounts of proteins were separated by 10% sodium dodecyl sulfate polyacrylamide gel electrophoresis, and then the proteins were transferred to polyvinylidene fluoride membranes using a power station (WSE-3500, ATTO, Tokyo, Japan). The membranes were incubated with diluted primary antibodies (Table S2) at 4 °C overnight. After washing three times with tris buffered saline (TBS) containing 0.1% Tween 20, the membranes were incubated with secondary antibodies for 2 h at room temperature. Finally, the membranes were developed by enhanced chemiluminescence using LAS-4000s (GE Healthcare, Chicago, IL, USA).

### 4.6. Propidium Iodide (PI) Staining

The deparaffinized and rehydrated slides were incubated with the PI solution and rinsed three times with PBS. The rinsed sections were incubated with 4′,6-diamino-2-phenylindole solution, rinsed with PBS, and mounted with a coverslip using Vectashield solution (Vector Laboratories, Burlingame, CA, USA). Fluorescence was detected by a confocal microscope (LSM 710; Carl Zeiss, Germany, Oberkochen, Germany).

### 4.7. Oil Red O (ORO) Staining for Hepatic Lipid Accumulation Measurement

The frozen liver specimens were sectioned at 10 µm and stained with ORO to analyze lipid deposition in hepatocytes. The frozen sections were rinsed with distilled water. After air-drying for a few minutes, the sections were soaked in 1,2-propanediol (Sigma-Aldrich St. Louis, MO, USA) for 5 min and stained with pre-warmed ORO for 10 min at 60 °C. Then, the tissues were differentiated in 85% 1,2-propanediol and rinsed with distilled water. The slides were mounted with a coverslip using glycerin (Sigma-Aldrich, St. Louis, MO, USA). The images were visualized by light microscopy (Olympus Optical Co., Tokyo, Japan), and quantification of the intensity of the red color was performed using ImageJ software 1.53j (National Institutes of Health, Bethesda, MD, USA).

### 4.8. Measurement of Triglycerides and Free Fatty Acids

The absolute concentrations of the liver protein samples of triglycerides (Abcam, Cambridge, UK) and free fatty acids (Abcam, Cambridge, UK) were determined using enzyme-linked immunosorbent assay (ELISA) kits. Absorbance was measured at 450 nm using an ELISA plate reader (Molecular Devices, N 1st. St San Jose, CA, USA).

### 4.9. Hematoxylin and Eosin (H&E) Staining for Histological Measurement

The livers were collected, fixed, and embedded in paraffin. The tissue sections were stained with H&E for the histological analysis of NAFLD activity. The NAFLD activity score was measured in a blinded manner according to the following criteria [27,28,29]: (1) Hepatic steatosis: score 0, <5% (none); score 1, 5% to 33% (mild); score 2, 34% to 66% (moderate); and score 3, >66% (severe). (2) Lobular inflammation: score 0, none; score 1, <2 foci/200× field; score 2, 2 to 4 foci/200× field; and score 3, >4 foci/200× field. (3) Hepatocellular ballooning: score 0, none; score 1, several; and score 2, large number. All images were acquired using light microscopy (Olympus Optical Co., Tokyo, Japan). At least three sections for each liver paraffine tissue were analyzed, and the average of these measurements was taken for analysis [27,28,29].

### 4.10. Statistical Analysis

The non-parametric tests were used in this study. The Kruskal–Wallis test was used to determine the significance of differences among the six groups. If a significant difference was confirmed by the Kruskal–Wallis test, multiple comparison was used with the Mann–Whitney U test. Experiments were performed in triplicate per animal, the results are presented as the mean ± standard deviation (SD), and statistical significance was accepted for p < 0.05. The analysis was conducted using SPSS version 22 (IBM Co., Armonk, NY, USA).

## 5. Conclusions

In conclusion, this study showed the beneficial effects of ECE or DK, which attenuated HFD-induced NAFLD by decreasing the NLRP3 inflammasome and pyroptosis.

## Supplementary Materials

The following are available online at https://www.mdpi.com/article/10.3390/md19060318/s1, Table S1: List of primers for qRT-PCR, Table S2: List of antibodies for immunohistochemistry and immunoblotting.
