# Supplementary material for: Attenuating Effects of Dieckol on High-Fat Diet-Induced Nonalcoholic Fatty Liver Disease by Decreasing the NLRP3 Inflammasome and Pyroptosis
Source: Mar Drugs. 2021 May 30;19(6):318. doi: 10.3390/md19060318 (PMC8227003; doi:10.3390/md19060318)
Supplement: Supplementary file 1 [file marinedrugs-19-00318-s001.zip › marinedrugs-1224243-supplementary.pdf]

Table S1. List of primers for qRT-PCR

Gene	Primers	
β-actin	Forward	5'-CCGTAAAGACCTCTATGCCAAC-3′
	Reverse	5'-GCAGTAATCTCCTTCTGCATCC-3′
HMGB1	Forward	5'-ACAAGCAGCCCTATGAGAAGAA-3′
	Reverse	5'-CTGCATCAGGTTTTCCTTTAGC-3′
TLR4	Forward	5'-ATTCAGAGCCGTTGGTGTATCT-3′
	Reverse	5'-TCAAGGACAATGAAGATGATGC-3′
NF-κB	Forward	5'-AGAACTGACTCTATGCCCGTGT-3′
	Reverse	5'-CATCTGTTGAGTTGGAGCTGAC-3′
FASN	Forward	5'-CTCTGGTGGTATCCACATCTCA-3′
	Reverse	5'-GTGAAAACGAACTTTTCCAAGG-3′
SREBP2	Forward	5'-AGGACTTAGTCATGGGGACAGA-3′
	Reverse	5'-TGACCTGCTGCAGATATTTGAT-3′
PPARα	Forward	5'-GTGTATGAAGCCATCTTCACGA-3′
	Reverse	5'-TAAGGAACTCGCGTGTGATAAA-3′
CPT1A	Forward	5'-TCTCTGCTGCATGGTAGATGTT-3′
	Reverse	5'-GCTCTGCGTTTATGCCTATCTT-3′
PPAR-γ	Forward	5′-TTCGCTGATGCACTGCCTAT-3′
	Reverse	5′-GGCATTGTGAGACATCCCCA-3′
FABP4	Forward	5′-CGATGAAATCACCGCAGACG-3′
	Reverse	5′-ACTCTTGTGGAAGTCACGCC-3′
ATGL	Forward	5′-CACGTCACCTGTGCCTTACT-3′
	Reverse	5′-ACGGCTGAGCAACTCTAACC-3′
HSL	Forward	5′-GGTGACACTCGCAGAAGACA-3′
	Reverse	5′-GCATCTCAAAGGCCTCAGGT-3′

Table S2. List of antibodies for immunohistochemistry and immunoblotting.

Antigen (host)	Company	Dilution rate (immunohistochemistry)	Dilution rate (immunoblotting)
ASC (mouse)	Santa cruz biotechnology	1:100	
NLRP3 (rabbit)	Abcam	1:100	
HMGB1 (rabbit)	Abcam	1:1000	
TLR4 (mouse)	Novus biologicals	1:200	
NF-κB (rabbit)	Cell signaling	1:500	
Caspase1(mouse)	Santa cruz biotechnology		1:1,000
Cleaved caspase1 (rabbit)	Cell signaling		1:1,000
Gasdermin D (rabbit)	Cell signaling		1:1,000
Cleaved gasdermin D (rabbit)	Cell signaling		1:1,000
β-actin (rabbit)	Cell signaling		1:1,000
